# A Case of Juvenile Polyposis Syndrome in a 13-year-old: A Case Report

**DOI:** 10.31729/jnma.8073

**Published:** 2023-03-31

**Authors:** Abashesh Bhandari, Bhupendra Kumar Basnet, Ashlesha Chaudhary, Aashutosh Chaudhary

**Affiliations:** 1 Department of Internal Medicine, Nepal Medical College and Teaching Hospital, Jorpati, Kathmandu, Nepal; 2 Department of Gastroenterology, Helping Hands Community Hospital, Chabahil, Kathmandu, Nepal; 3 Nepal Medical College, and Teaching Hospital, Jorpati, Kathmandu, Nepal; 4 Kathmandu University School of Medical Sciences, Dhuiikhel, Kavre, Nepal

**Keywords:** *case reports*, *children*, *juvenile polyposis syndrome*

## Abstract

Juvenile polyposis syndrome is an autosomal dominant syndrome characterised by hamartomatous polyps in the gastrointestinal tract and has a high risk for colon carcinoma. This case explores the presentation of multiple polyps throughout the gastrointestinal tract, located in the stomach, proximal duodenum, colon, rectum and up to the anal canal. The locations and number of these polyps themselves were not typical and the histopathological studies suggested the condition to be an inflammatory fibroid polyp, which is a rare, benign and solitary neoplasm. Prompt and accurate diagnostic modality remains the keystone in the identification and management of such condition which was a limitation in this case as the patient was lost to follow up before a definitive diagnosis was made.

## INTRODUCTION

The gastrointestinal polyposis syndromes represent a unique set of inherited syndromes that may predispose to gastrointestinal cancers and have rather complex presentations.^1-3^ The management of these patients should depend primarily on their clinical diagnosis as their phenotypical features may vary largely among patients regardless of their germline mutation.^4^ Our case studies the occurrence of multiple polyps throughout the gastrointestinal tract, located in the stomach, proximal duodenum, colon, rectum and up to the anal canal, depicting a rare presentation as the location as well as the number was not typical.

## CASE REPORT

A 13-year-old male was referred for evaluation of black tarry stool for 4 days and two episodes of vomiting which was non-bilious and non-blood mixed. His past medical history and family history were insignificant. His systemic examination including per rectal examination was normal. Proctoscopy was done which did not show the presence of haemorrhoids or ulcers. His initial workup on presentation which included blood investigations, stool routine examination and stool for occult blood were within normal limits.

**Figure 1:**
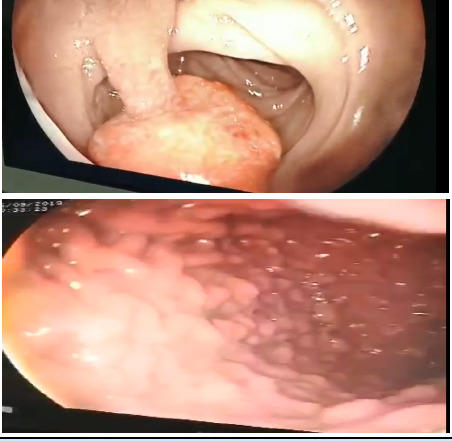
Multiple polyps of variable sizes as seen on upper gastrointestinal endoscopy.

The patient was then planned for contrast-enhanced computed tomography (CECT) abdomen with contrast which showed multiple prominent mucosal folds which were pedunculated in the mucosa of the stomach and the proximal duodenum. Based on these findings, an upper gastrointestinal endoscopy was done the next day, which showed the presence of multiple polyps in the fundus, body, and antrum extending into the visible part of the duodenum (Figure 1).

**Figure 2:**
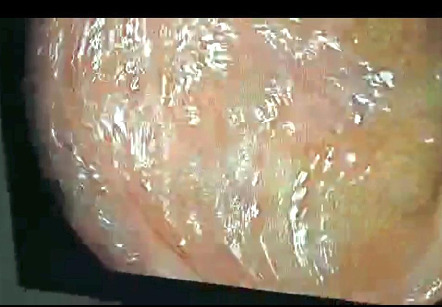
Multiple polyps seen in splenic flexure.

**Figure 3:**
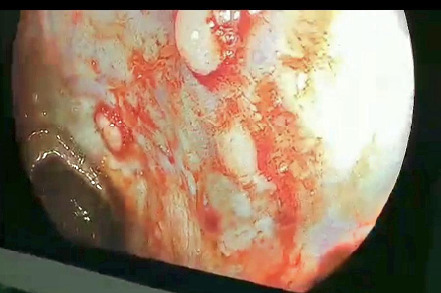
Multiple polyps seen in sigmoid colon.

After this, a colonoscopy was done which showed multiple polyps of variable sizes over the entire rectum up to the anal canal. Splenic flexure, hepatic flexure as well as the ascending and transverse colon showed the presence of multiple polyps (Figure 2, 3). Multiple biopsies were taken and sent for histopathology evaluation which showed fragments of colonic mucosa with moderate infiltration of plasma cells, and lymphocytes and marked eosinophilic infiltration without atypical cells. These findings were suggestive of inflammatory polyps and were negative for malignancy.

The patient was suggested further workup including germline testing and management but the patient was lost to follow-up.

## DISCUSSION

This case explores the occurrence of multiple gastrointestinal polyps in the stomach, duodenum, splenic flexure, hepatic flexure as well as the ascending and transverse colon rectum up to the anal canal. The gastrointestinal polyposis syndromes, a predisposing factor for gastrointestinal cancers require genetical evaluation, especially in patients with a known family history of gastrointestinal cancers and polyposis, young individuals, patients with more than ten colonic adenomatous polyps or more than two hamartomatous polyps and multiple polyps aided by proper clinical assessment and histopathological study.^5^ However, genetic evaluation was not possible in our case as the patient was lost to follow-up genetic evaluation couldn't be done.

Juvenile polyposis syndrome (JPS) is an autosomal dominant syndrome characterized by multiple hamartomatous polyps which may predispose to a greater risk for cancer. These polyps usually begin to appear in the first decade of life. The primary sites include the colorectum (98%), stomach (14%), duodenum (7%), jejunum, and ileum (7%).^6,7^ At least one of the following criteria is required for the diagnosis of JPS after ruling out other polyposis syndrome,^6,8^ which include: 1) more than five juvenile polyps in the colorectum, 2) multiple juvenile polyps in other parts of the gastrointestinal tract and 3) any number of juvenile polyps in a person with a known family history of juvenile polyps.

Inflammatory fibroid polyp (IFP) is a rare benign solitary mesenchymal lesion of the gastrointestinal tract which can occur in any age group (peak incidence is between the sixth and seventh decades of life. This condition is slightly more common in males. The most common location is the stomach (66-75%), followed by the small intestine, predominantly ileum (18-23%), colon and rectum (4-7%), gallbladder (1%), oesophagus (1%) and appendix.^1^ IFP of the duodenum is a remarkably rare occurrence.^9^ The histopathological report of our case suggested inflammatory fibroid polyps too but the confirmation could not be made. IFPs tend to be solitary lesions in the elderly and their occurrence in the duodenum and colon is not common which the case in this patient was.

Duodenal polyps are rare entity.^10^ Incidental diagnoses of duodenal polyps seem to be increasing, due to the wide use of esophagogastroduodenoscopy. Although duodenal polyps may be pedunculated in nature, these polyps are sessile and small, non-neoplastic. Inflammatory polyps contain ectopic gastric mucosa and are frequently present in the duodenum.^10^

Given the rarity of the different spectrums of this condition, the diagnosis is likely to be missed, especially during the initial presentation. However, lack of financial resources can be a dilemma in resource-limited countries such as ours for the management of such patients as patients tend to avoid follow-up as happened in this case. Proper clinical history and meticulous workup can aid in the diagnosis of gastrointestinal polyposis syndromes, supported by genetic evaluation and could possibly lead to a reduction in mortality and morbidity in such patients in deprived populations as well.
